# Glycomic Analysis Reveals That Sialyltransferase Inhibition Is Involved in the Antiviral Effects of Arbidol

**DOI:** 10.1128/jvi.02141-21

**Published:** 2022-03-23

**Authors:** Yue Kang, Zhi-Tong Mai, Lee-Fong Yau, Run-Feng Li, Tian-Tian Tong, Chun-Guang Yang, Ka-Man Chan, Zhi-Hong Jiang, Yutao Wang, Zi-Feng Yang, Jing-Rong Wang

**Affiliations:** a State Key Laboratory of Quality Research in Chinese Medicine, Macau Institute for Applied Research in Medicine and Health, Macau University of Science and Technology, Macau, China; b State Key Laboratory of Respiratory Disease, National Clinical Research Center for Respiratory Disease, Guangzhou Institute of Respiratory Health, The First Affiliated Hospital of Guangzhou Medical University, Guangzhou, China; c KingMed Virology Diagnosis & Translational Center, Guangzhou, China; Lerner Research Institute, Cleveland Clinic

**Keywords:** influenza virus, arbidol, sialic acid receptor, sialyltransferase, glycomic

## Abstract

Due to the high mutation rate of influenza virus and the rapid increase of drug resistance, it is imperative to discover host-targeting antiviral agents with broad-spectrum antiviral activity. Considering the discrepancy between the urgent demand of antiviral drugs during an influenza pandemic and the long-term process of drug discovery and development, it is feasible to explore host-based antiviral agents and strategies from antiviral drugs on the market. In the current study, the antiviral mechanism of arbidol (ARB), a broad-spectrum antiviral drug with potent activity at early stages of viral replication, was investigated from the aspect of hemagglutinin (HA) receptors of host cells. *N-*glycans that act as the potential binding receptors of HA on 16-human bronchial epithelial (16-HBE) cells were comprehensively profiled for the first time by using an in-depth glycomic approach based on TiO_2_-PGC chip-Q-TOF MS. Their relative levels upon the treatment of ARB and virus were carefully examined by employing an ultra-high sensitive qualitative method based on Chip LC-QQQ MS, showing that ARB treatment led to significant and extensive decrease of sialic acid (SA)-linked *N-*glycans (SA receptors), and thereby impaired the virus utilization on SA receptors for rolling and entry. The SA-decreasing effect of ARB was demonstrated to result from its inhibitory effect on sialyltransferases (ST), ST3GAL4 and ST6GAL1 of 16-HBE cells. Silence of STs, natural ST inhibitors, as well as sialidase treatment of 16-HBE cells, resulted in similar potent antiviral activity, whereas ST-inducing agent led to the diminished antiviral effect of ARB. These observations collectively suggesting the involvement of ST inhibition in the antiviral effect of ARB.

**IMPORTANCE** This study revealed, for the first time, that ST inhibition and the resulted destruction of SA receptors of host cells may be an underlying mechanism for the antiviral activity of ARB. ST inhibition has been proposed as a novel host-targeting antiviral approach recently and several compounds are currently under exploration. ARB is the first antiviral drug on the market that was found to possess ST inhibiting function. This will provide crucial evidence for the clinical usages of ARB, such as in combination with neuraminidase (NA) inhibitors to exert optimized antiviral effect, etc. More importantly, as an agent that can inhibit the expression of STs, ARB can serve as a novel lead compound for the discovery and development of host-targeting antiviral drugs.

## INTRODUCTION

Globally, the high morbidity and mortality of influenza virus infections have set off the alarm for a global pandemic, thus prompting extensive investigations on antiviral approaches (1). Currently, a number of antiviral drugs based on different mechanism of actions have been developed (2–5); however, efficacy of these drugs has been jeopardized by the emergence of drug resistance (6), which has pinpointed an urgent need for developing new approaches for virus prevention. Due to the high mutation rate of influenza virus and the rapid increase of drug resistance against virus-targeting drugs, targeting hosts might represent an alternative and attractive direction for overcoming drug resistance (7) and achieving broad-spectrum antiviral effect.

The virus invasion was first initiated by the attachment of viral hemagglutinin (HA) to receptors containing *N*-glycans with terminal sialic acids (SA receptors) on the host cell surface. *α*2,3- and *α*2,6-linkage are the two major linkages between sialic acid (SA) and the penultimate galactose residue (8–10). In general, human influenza viruses preferentially recognize *α*2,6-SA receptors while avian influenza viruses preferentially bind to *α*2,3-SA receptors (11, 12). Due to the crucial role of the HA binding to its appropriate SA receptors, targeting SA receptors might show merits on lowering drug resistance and represents a potential therapeutic option (13). Recently, DAS181, a novel sialidase fusion protein (7, 14), was demonstrated to inhibit human and avian influenza virus infection by decreasing sialylation of both *α*2,3- and *α*2,6-SA receptors in human lung tissues, suggesting targeting SA receptors as a promising antiviral approach (15). However, the strategy for specific targeting virus-relevant SA receptors remains unclear. Meanwhile, structural preferences of virus binding are extremely complicated (16, 17), making it sophisticated to modify SA receptors simply based on virus binding tropism (18, 19). Moreover, small molecules that target SA receptors are still lacking. Taking into consideration the discrepancy between the urgent demand of antiviral drugs during the influenza pandemic and the long-term process of drug discovery and the development, exploring HA receptor-related activity and underlying mechanisms of antiviral drugs on the market would provide an feasible approach for the development of antiviral agent targeting HA receptors, and more importantly, may offer the public with an out-of-box antiviral agent that can target host cells during an influenza pandemic.

Arbidol (ARB) is a non-nucleoside antiviral drug developed by the Pharmaceutical Chemistry Research Center of the Former Soviet Union. It is commonly used for the treatment of influenza A and B (20, 21). Unlike the antiviral drugs currently available on the market, i.e., neuraminidase (NA) inhibitors such as zanamivir and oseltamivir, as well as matrix protein 2 (M2) ion channel inhibitors such as amantadine and rimantadine, ARB is a broad-spectrum antiviral drug that possesses dual impact on both virus and host, and also exhibits low viral resistance (20, 22, 23). Although it has been well-documented that ARB prevents virus invasion at different steps of the virus infection cycle, especially at early stages of virus replication, by inhibiting the fusion of virus and host cell, or by preventing pH-induced transitioning of HA into its functional fusogenic state (20, 22, 23), its specific target has not been fully illustrated. Notably, ARB has been reported to exert the maximum antiviral activity when used before infection (20), indicating its potential effect on regulating host-based factors, but the underlying mechanisms were not clear. Considering that ARB impedes virus infection at the entry step, and that the process of virus entry is closely associated with the glycans acting as HA receptors on the host cell surface, as well as that ARB is structurally classified as an alkaloid that was reported as a glycan regulator, we hypothesized that the antiviral activity of ARB may be associated with host cell surface glycans.

Given that more than 93% cell surface SAs are derived from *N-*glycans on glycoproteins, and the *N-*glycans in the human respiratory tract possess extensive structural diversification and are more closely relevant to influenza virus binding (13–15), we performed a comprehensive glycomic analysis on the *N-*glycans of 16-human bronchial epithelial (16-HBE) cells employing an in-depth glycomic approach established in our lab. We further examined the influence of ARB on the *N-*glycans of 16-HBE cells to explore the host-targeting mechanisms of ARB.

## RESULTS

### Comprehensive profiling and quantitative analysis of *N*-glycans in 16-HBE cells.

**FIG 1:**
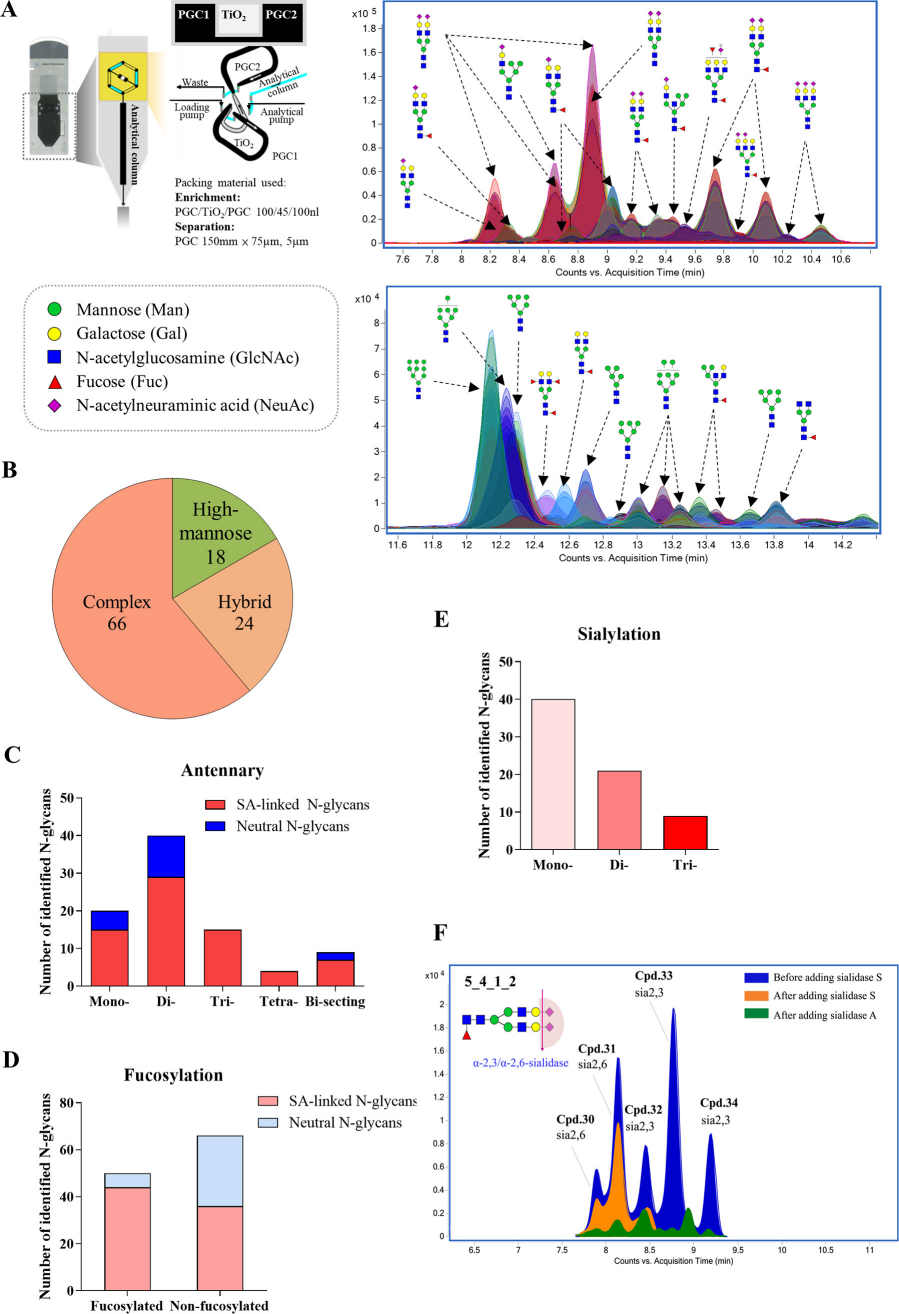
Comprehensive profiling of *N*-glycans in 16-HBE cells. (A) The overlaid extracted compound chromatograms (ECC) of 108 *N-*glycans identified in 16-HBE cells by using TiO_2_-PGC chip-Q-TOF-MS method. Numbers of identified *N-*glycans in each class of glycan are classified based on (B) high mannose/hybrid/complex, (C) different numbers of antennary, (D) with or without fucose, (E) different numbers of SAs. (F) ECC of 5_4_1_2 before and after sialidase S or sialidase A treatment.

*N-*glycans of 16-HBE cells were comprehensively profiled for the first time by using a well-established TiO_2_-PGC chip-Q-TOF-MS method established in our lab (24, 25). For the identification of *N-*glycans based on MS data, a database containing around 3,800 *N-*glycans was built on the basis of the knowledge of mammalian *N-*glycan biosynthesis for the identification. At the MS level, *N-*glycans of 16-HBE cells were primarily assigned, and the assigned structures were then verified based on high-resolution MS/MS data. As a result, 70 SA-linked *N-*glycans derived from 24 compositions and 38 neutral *N-*glycans derived from 16 compositions were identified in 16-HBE cells (Fig. 1A and Table S1).

The identified *N-*glycans were classified into three different types, in which complex was the predominant type, while high-mannose and hybrid type were less abundant (Fig. 1B). Among the identified *N-*glycans, di-antennary type occupied the major portion, followed by mono-, tri-, bisecting, and tetra-antennary type (Fig. 1C). Additionally, the number of identified non-fucosylated *N-*glycans was higher than the fucosylated types, and the majority of fucosylated *N-*glycans were sialylated (Fig. 1D). The degree of sialylation varied from one to three, among which mono-sialylated *N-*glycans accounted for the majority, while tri-sialylated *N-*glycans accounted for the least (Fig. 1E). The SA linkages were further confirmed by using sialidase reactions; this can be exemplified by 5_4_1_2 (Fig. 1F). By using this strategies, 61 SA-linked *N-*glycans were identified to possess *α*2,6-linkage and only nine possess *α*2,3-linkage (Table S1).

**FIG 2:**
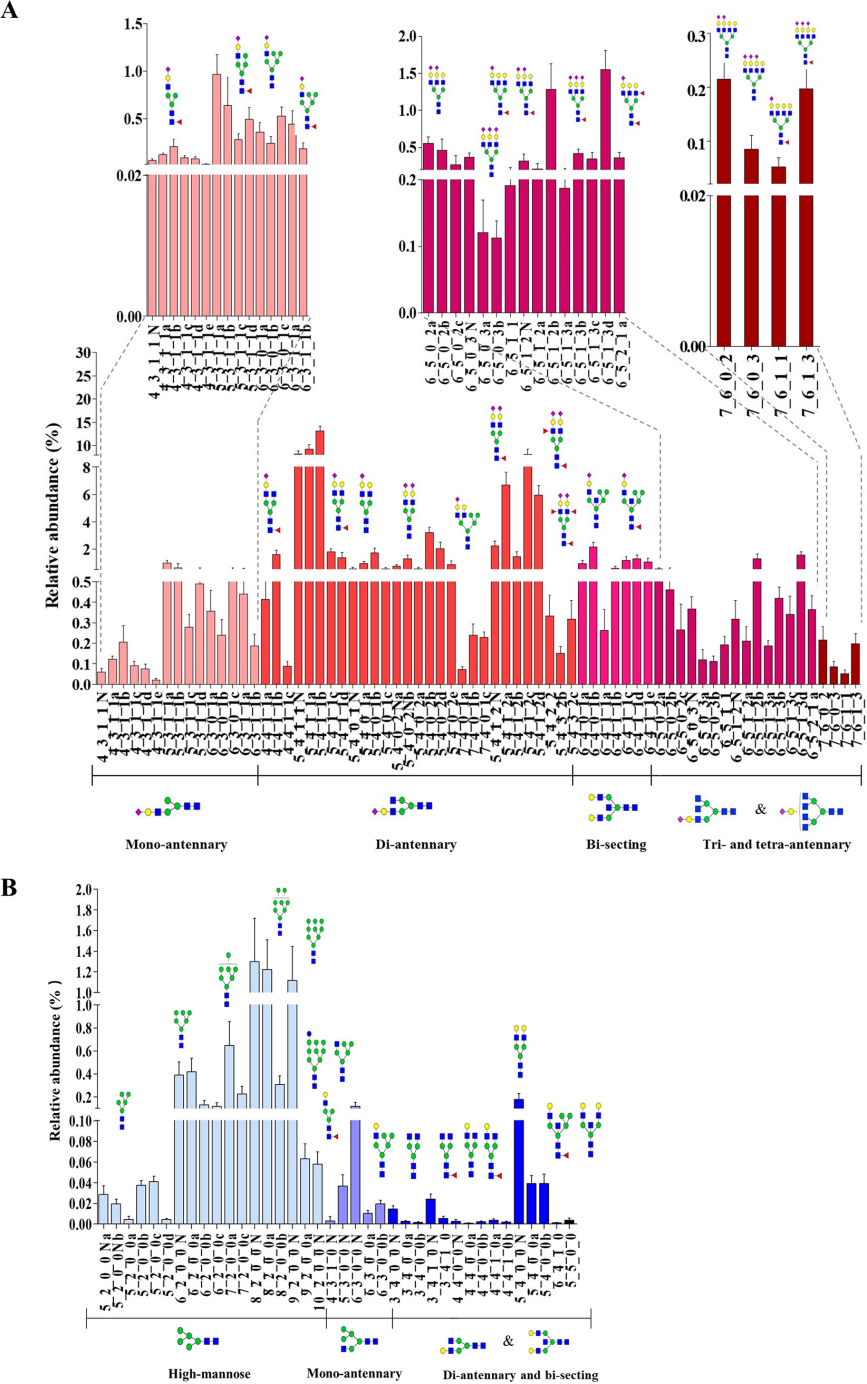
The quantitative profiles of (A) SA-linked *N*-glycans (B) and neutral *N-*glycans in 16-HBE cells by using TiO_2_-PGC chip-QQQ-MS in MRM mode. Data were shown as mean ± SD of three dependent experiments. For the full name of each *N*-glycan refer to Table S1.

The identified *N-*glycans in 16-HBE cells were then quantified by using TiO_2_-PGC chip-QQQ-MS in multiple reaction monitoring (MRM) mode (24). As shown in Fig. 2A, SA-linked *N-*glycans were significantly more abundant than neutral *N-*glycans, as evidenced by over 90% relative abundance of the SA-linked *N-*glycans. Among SA-linked *N-*glycans, di-antennary type accounted for the most abundant species, and 5_4_1_1 and 5_4_1_2 were the most dominant structures (more than 8%) (Fig. 2A). On the other hand, the relative abundance of all individual neutral *N-*glycan was less than 2%. Among neutral *N*-glycans, high-mannose type occupied the major portion, in which 8_2_0_0 and 9_2_0_0 had the highest relative abundance (more than 1%) (Fig. 2B).

### Cytotoxicity and antiviral activity of ARB.

**FIG 3:**
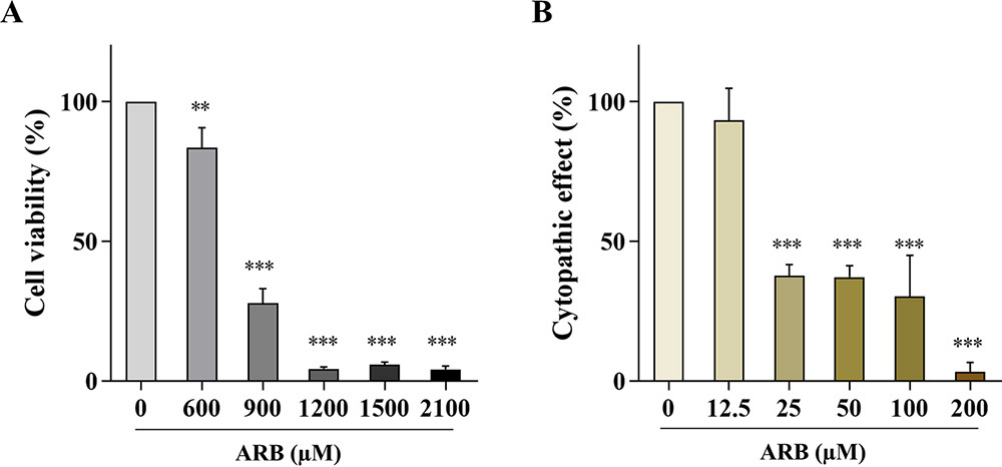
Cytotoxicity and antiviral activity of ARB. (A) 16-HBE cells were treated with ARB (0, 600, 900, 1,200, 1,500, 2,100 μM) for 48 h, cellular toxicity of ARB on 16-HBE cells was evaluated by using MTT assay. (B) Inhibitory effect of ARB against influenza virus A/Puerto Rico/8/1934 (H1N1) in 16-HBE cells (MOI = 0.01). *, *P < *0.05; **, *P < *0.01; ***, *P < *0.001 compared with the control groups.

The cell viability of ARB in 16-HBE cells was evaluated by using 3-(4,5-Dimethylthiazol-2-yl)-2,5-diphenyltetrazolium bromide (MTT) assay, and the TC_50_ of ARB was determined to be 775.80 ± 2.04 μM. As shown in Fig. 3A, ARB didn’t show significant cytotoxicity toward 16-HBE cells under concentrations up to 600 μM. The concentrations of ARB used in antiviral study were therefore designed as 12.5 to 200 μM. The antiviral activity of ARB against PR8 infection (multiplicity of infection [MOI] = 0.01) in 16-HBE cells was evaluated using cytopathic effect (CPE) inhibition assay; the IC_50_ value was determined to be 51.50 ± 1.06 μM (Fig. 3B), and the selectivity index (SI) was calculated as 15.06. It is the first time that the antiviral effect of ARB against PR8 virus on 16-HBE cells was assayed. It should be noted that ARB was added prior to virus infection. After 4 h of incubation, the drug solution was removed and PR8 virus was then inoculated. Such pretreatment procedure clearly showed that the observed antiviral activity of ARB should be ascribed to the host cells, but not due to direct viricidal activity toward virus. This result supported further exploration of the host-targeting mechanisms of ARB.

### Alteration of *N*-glycan profile in 16-HBE cells upon PR8 infection.

**FIG 4:**
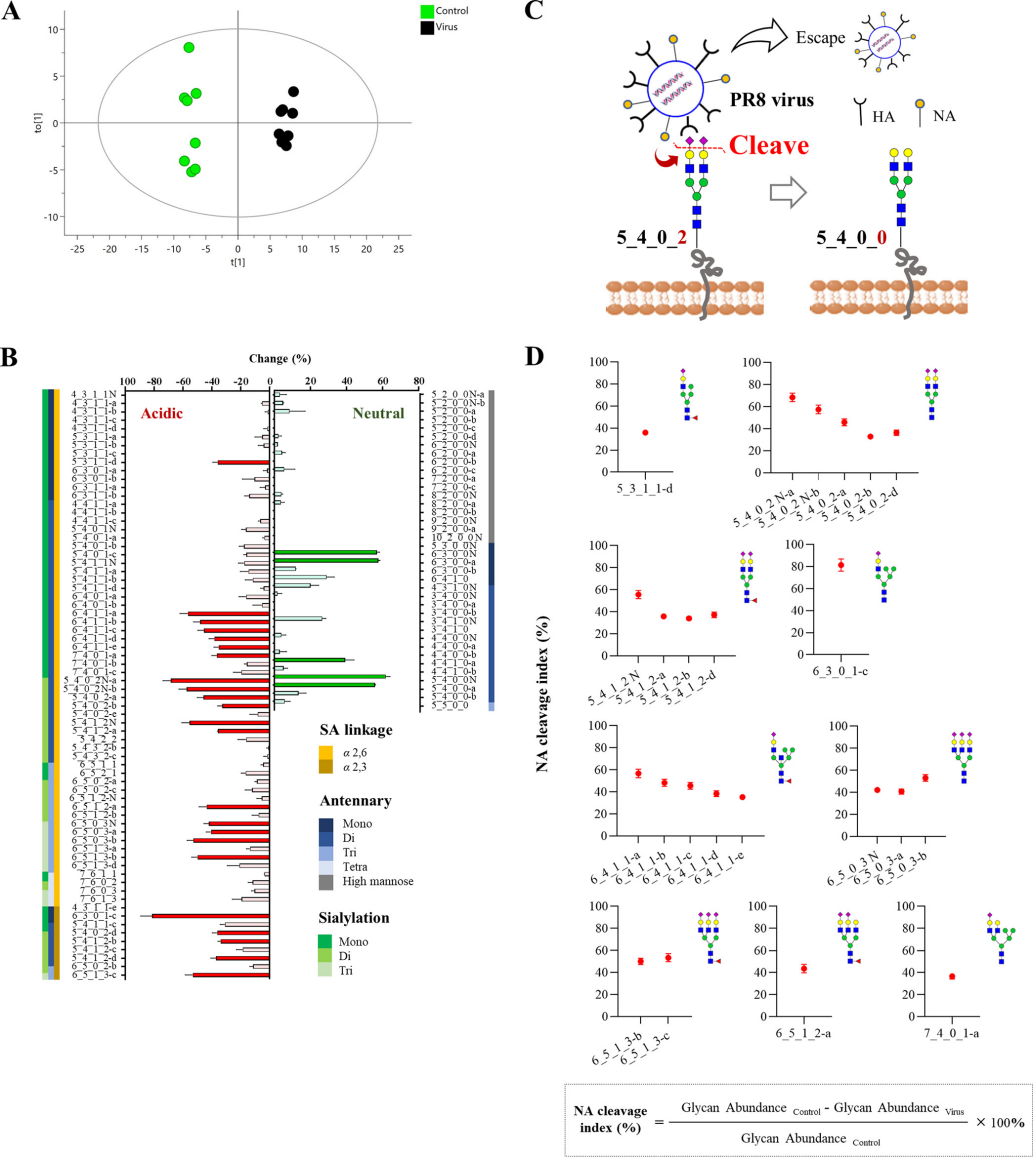
Altered levels of *N*-glycans in 16-HBE cells after PR8 virus infection. (A) The OPLS-DA score plot for discrimination of control group (*n *= 8) and virus group (*n *= 8) (*R^2^X *= 0.666, *R^2^Y *= 0.992, *Q^2^* = 0.974). (B) The histogram showing the change of *N*-glycan level in 16-HBE cells after incubation with PR8 virus (MOI = 0.01) for 2 h at 4°C. Glycan structures were sorted based on SA linkage, numbers of antennary, and SAs. The column for biomarkers (VIP ≥ 1) were marked in dark color. (C) Schematic representation of the involvement of NA cleavage of virus to the downregulation of SA-linked *N*-glycans and upregulation of neutral *N*-glycans. (D) The NA cleavage index of all SA-linked *N-*glycan biomarkers were calculated by (Glycan abundance in control – Glycan abundance after infection)/Glycan abundance in control × 100%.

To explore the influence of PR8 infection on the *N*-glycan expression in 16-HBE cells, quantitative glycomic analyses of control group and virus group were performed. Multivariate analysis of the obtained glycomic data was carried out to observe the overall alteration of *N-*glycans upon PR8 infection. As shown in Fig. 4A, the OPLS-DA score plot demonstrated clear separation of the control and virus groups (*R^2^X *= 0.666, *R^2^Y *= 0.992, *Q^2^* = 0.974), suggesting that the *N-*glycan profile in 16-HBE cells significantly altered upon PR8 infection. Variable importance plot (VIP) value ≥ 1 were considered as the significant biomarkers contributing to the discrimination between the control and the virus groups. A total of 28 *N*-glycans including 23 SA-linked *N*-glycans and five neutral *N*-glycans were identified as biomarkers. Among 23 SA-linked *N*-glycan biomarkers, 5_3_1_1, 5_4_0_2, 5_4_1_2, 6_3_0_1, 6_4_1_1, 6_5_0_3, 6_5_1_3, 6_5_1_2, 7_4_0_1 significantly decreased by 30% to 80% after PR8 infection. Five neutral *N*-glycan biomarkers 6_3_0_0, 4_4_1_0, 5_4_0_0 were significantly upregulated by 40% to 60% after PR8 infection (Fig. 4B).

Above results suggested clearly that SAs were depleted during PR8 infection. In general, during the early stages of virus replication (0 to 2 h), the virus continuously rolls and glides on the cell surface via a HA-receptor exchange mechanism (26, 27), during which virus HAs bind to SA receptors and NA protein cleave SAs, the density gradient of SA would promote virus rolling and gliding until the virus finds an appropriate entry receptor. Therefore, it can be speculated that the depletion of SAs should be resulted from the cleavage of SAs on host cell surface by virus NA protein during virus infection. The NA cleavage also led to the increase of neutral glycans as cleavage product, as exemplified in Fig. 4C, virus NA protein will cleave the terminal SAs of 5_4_0_2 and generate 5_4_0_0 during virus rolling.

As aforementioned, the 23 significantly decreased SA-linked *N-*glycans might be the decoy and entry receptors utilized by the virus for rolling and entry. Among the SA-linked *N-*glycans cleaved by virus NA protein, 18 possess *α*2,6-linkage and five possess *α*2,3-linkage. In addition, most of the downregulated *N-*glycans are di-antennary *N-*glycans terminated with one or two SAs and tri-antennary *N-*glycans terminated with three SAs (Fig. 4D).

In the early stage of the virus infection, cooperation of virus HA binding to SA decoys and virus NA cleaving SA receptors act as the motile machinery to promote viral motility and initiate infection (26, 27). Thus, the utilization capability on the SA receptors actually indicate the receptor-dependent-rolling and entry capacity of virus. The more active the virus movement and binding, the more SAs receptors would be cleaved. For better evaluating the virus utilization of SA receptors, we adopted a so-called “NA cleavage index,” which was calculated as the decreasing degree of SA-linked *N-*glycans upon virus infection. As shown in Fig. 4D, the NA cleavage index of those virus-utilized SA receptors are generally 30–80% upon virus infection.

### Influence of ARB on the *N*-glycans of 16-HBE cells.

**FIG 5:**
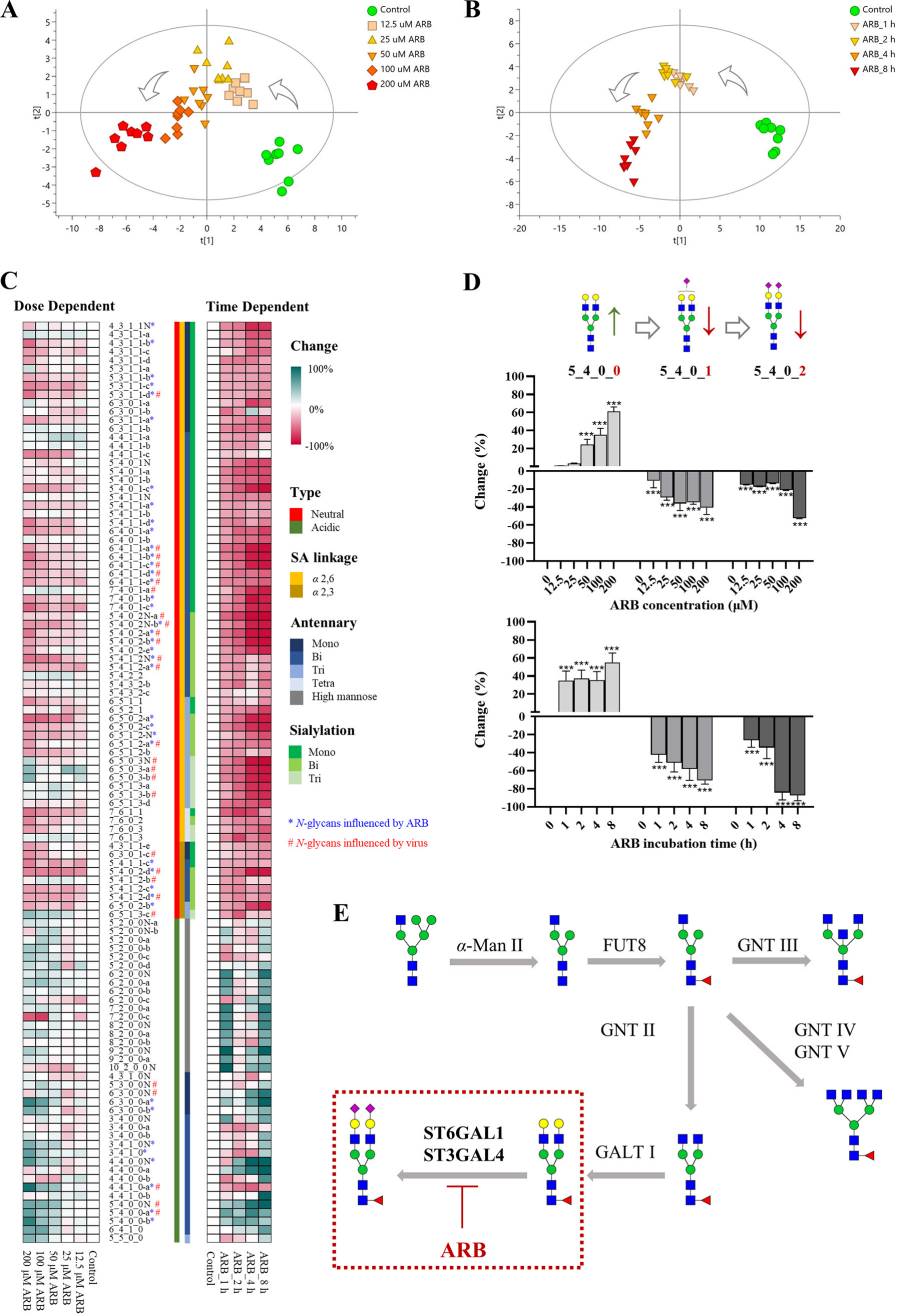
Altered levels of *N-*glycans in 16-HBE after ARB treatment. The OPLS-DA score plot for discrimination of (A) control group (*n *= 8) and dose-dependent ARB treatment groups (0, 12.5, 25, 50, 100, 200 μM, *n *= 8) (*R^2^X *= 0.677, *R^2^Y *= 0.800, *Q^2^* = 0.726), and (B) control groups (*n *= 8) and time-dependent ARB treatment groups (treated with 50 μM ARB for 1, 2, 4, 8 h, *n *= 8) (*R^2^X *= 0.711, *R^2^Y *= 0.898, *Q^2^* = 0.600). (C) The heatmap showing the change of *N*-glycan level in 16-HBE cells along with ARB treatment concentrations and treatment times. Glycan structures were sorted based on SA linkage, numbers of antennary, and SAs. The percentage changes were calculated by (Glycan abundance after ARB treatment – Glycan abundance in control)/Glycan abundance in control × 100%. The biomarkers related to ARB treatment and virus infection were labeled with an asterisk (*) and a pound sign (#), respectively. (D) Neutral *N*-glycan biomarkers (5_4_0_0) were upregulated concomitant with the downregulation of its corresponding sialylated species (5_4_0_2 and 5_4_0_1) in both a dose- and time-dependent manner. (E) Schematic representation of the involvement of ARB inhibition within the mammalian *N-*glycosylation processing pathways.

The *N*-glycan profiles of a control group and ARB groups with different concentrations (at 12.5, 25, 50, 100, 200 μM for 4 h treatment) and incubation times (at 50 μM for 1, 2, 4, 8 h treatment) were compared to view the effect of ARB on the expression of *N-*glycans. As shown in Fig. 5A, good visual separation was observed between the control group and the dose-dependent ARB treatment groups (*R^2^X *= 0.677, *R^2^Y *= 0.800, *Q^2^* = 0.726). ARB groups gradually shift away from the control group with the increase of ARB concentrations. A similar trend was observed for the control group and the time-dependent ARB treatment groups (*R^2^X *= 0.711, *R^2^Y *= 0.898, *Q^2^* = 0.600) (Fig. 5B). These results suggested dose- and time-dependent regulation of ARB on the *N-*glycan expression.

Heatmap was generated for better viewing the changes along with treatment of ARB with varied concentrations and treatment times. The results showed that ARB treatment led to extensive downregulation of SA-linked *N-*glycans in a dose- and time-dependent manner (Fig. 5C). A total of 32 SA-linked *N-*glycan biomarkers (VIP value ≥ 1) were recognized, including 5 *α*2,3- and 27 *α*2,6 SA-linked *N-*glycans. Most of these SA-linked *N-*glycan biomarkers were in di-antennary type, while 17 of them contained one SA and 15 of them contained two SAs. Among these ARB-regulated SA-linked *N-*glycan biomarkers, 14 SA-linked *N*-glycans were decoys and entry receptors utilized by virus for rolling and entry, implying that the virus utilization of their decoys and entry receptors might be impaired by SA-downregulating effect of ARB.

In addition, an interesting phenomenon observed for ARB treatment is, along with the decrease of SA-linked *N-*glycans, ARB treatment caused concomitant elevation of neutral *N-*glycans (Fig. 5C). Notably, structural relevance between the upregulated neutral *N-*glycans and downregulated SA-linked *N-*glycans was found. This can be exemplified in Fig. 5D; neutral *N*-glycan biomarkers 5_4_0_0 were significantly upregulated, while its corresponding sialylated species (e.g., 5_4_0_2 and 5_4_0_1) decreased significantly in both dose- and time-dependent manner, indicating structural transformation between the neutral and SA-linked *N-*glycans might be changed by ARB.

In the biosynthesis pathway of *N-*glycans, the neutral *N-*glycans of complex type are generally sialylated by sialyltransferase (ST) to generate corresponding mono-, di-, or multiple antennary SA-linked *N-*glycans (Fig. 5E). The increased neutral *N-*glycans upon ARB treatment are exactly the biosynthetic precursors of those decreased SA-linked *N-*glycan biomarkers, which clearly indicated an inhibited sialylation of the *N-*glycans of 16-HBE cells by ARB treatment. As STs are enzymes responsible for the sialylation of *N-*glycans, it is very likely that ARB inhibited expression of this enzyme, and led to the accumulation of precursor neutral *N-*glycans and decreased production of sialylated *N-*glycans.

### ARB exhibited its antiviral activity through *N-*glycan regulation.

Given that ARB can downregulate a number of SA-linked *N-*glycans of 16-HBE cells, and some of these downregulated SA-linked *N-*glycans are employed as decoys and entry receptors for virus rolling and entry, we then need to know whether such downregulation would impair the virus utilization on SA-linked *N-*glycans. This would be crucial evidence for understanding the role of SA-linked *N-*glycans in ARB’s antiviral activity. We therefore examined the glycomic changes of ARB pretreatment plus virus infection.

**FIG 6:**
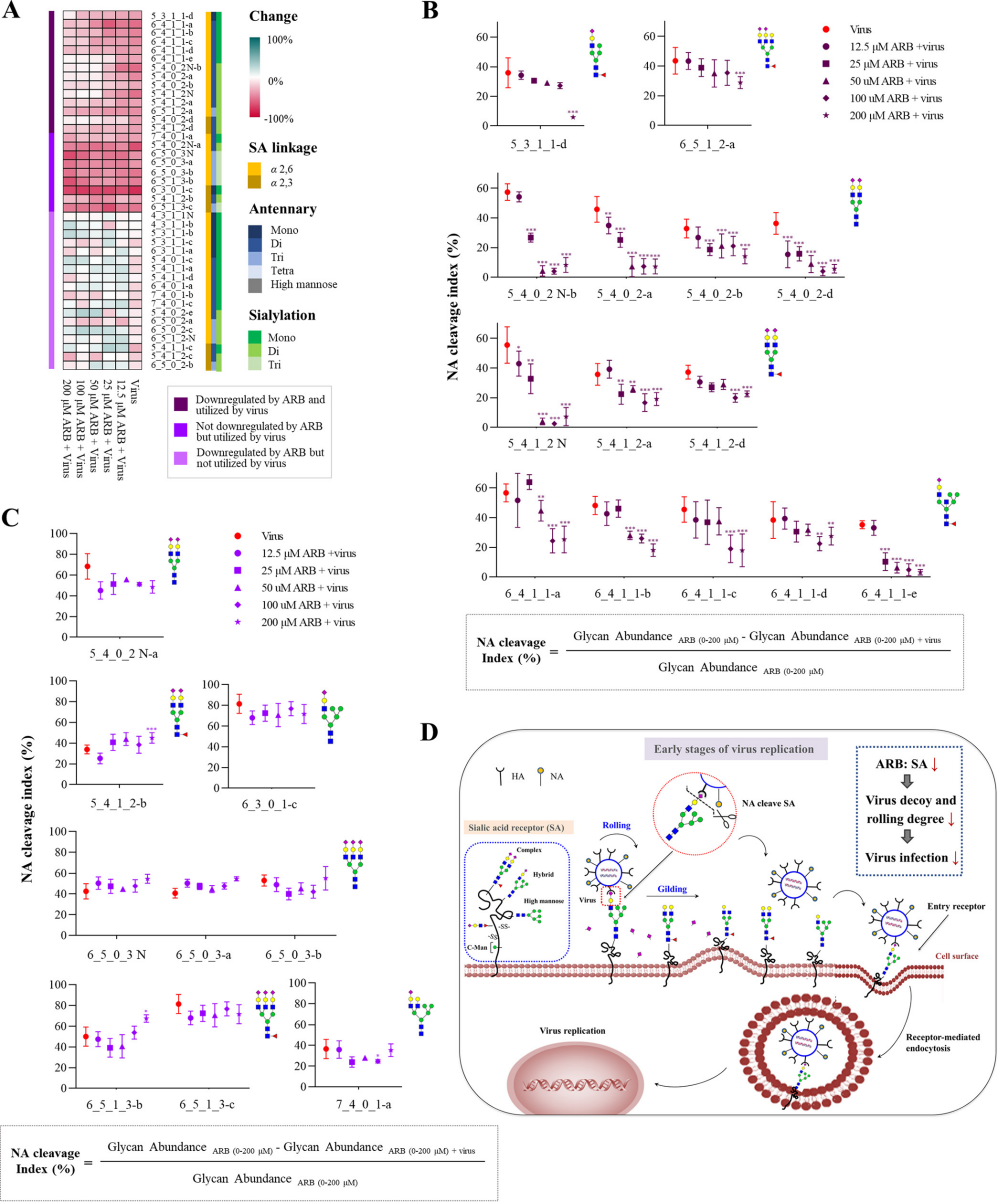
Altered levels of *N-*glycans in 16-HBE cells upon ARB pretreatment plus virus infection. (A) The heatmap showing the changes of individual *N*-glycan affected by ARB or/and PR8 in ARB+virus group compared with respective ARB groups. Glycan structures were sorted based on SA linkage, numbers of antennary and SAs. (B) The NA cleavage index of 14 SA-linked *N-*glycans downregulated by ARB and utilized by virus. *, *P < *0.05; **, *P < *0.01; ***, *P < *0.001 compared with the virus group. (C) The NA cleavage index of nine SA-linked *N-*glycans not downregulated by ARB but utilized by virus. (D) Schematic representation of early stages of virus replication (0 to 2 h) that exhibited the potential antiviral activity of ARB.

First, heatmap was generated to visualize the levels of SA-linked *N*-glycans affected by ARB or/and PR8 virus. As we aimed to examine the ARB impact on the utilization of SA receptors by virus, the changes (%) of individual *N*-glycan in ARB+virus group versus respective ARB group therefore showed in the heatmap (Fig. 6A). For the SA-linked *N*-glycans both downregulated by ARB and utilized by virus, their changes owing to virus infection obviously declined along with the increasing concentrations of ARB, suggesting that the virus utilization on these glycans are impacted by ARB. For those SA-linked *N*-glycans that were not downregulated by ARB but utilized by virus, their changes (%) remained at a level similar to sole virus infection for all ARB concentration groups, showing that virus engagement on these *N-*glycans were not influenced by ARB, For those SA-linked *N*-glycans that were downregulated by ARB but not utilized by virus, ARB+virus treatment didn’t cause obvious changes compared to corresponding ARB group, indicating that they were not employed by virus even when the decoy-receptors were downregulated.

For better interpretation, the NA cleavage index of the ARB-regulated and virus-utilized *N-*glycans are additionally shown in Fig. 6B. It can be seen that the cleavage index of 14 virus-utilized SA-linked *N*-glycans are generally 35% to 60% upon virus infection alone. After pretreatment with ARB, NA cleavage index of these receptors decreased in a dose-dependent manner. The index of receptors 5_4_0_2, 5_4_1_2 decreased remarkably to less than 30%, and even low to < 5% under 200 μM, suggesting that receptor-dependent-rolling and entry capacity of the virus are significantly retarded upon the downregulation of SA receptors by ARB. Meanwhile, NA cleavage index of nine SA-linked *N-*glycans that were not downregulated by ARB maintained a high level in ARB pretreatment groups, indicating that these *N-*glycans can still act as receptors for virus rolling and entry (Fig. 6C).

When virus movement and binding increases, SA cleavage also increases (26, 27). Therefore, the NA cleavage index can indicate virus utilization capability on the SA-linked *N-*glycans, and further indicate the receptor-dependent-rolling and entry capacity of virus. The above results showed that downregulation of SA-linked *N-*glycans by ARB significantly impaired the virus utilization on SA-linked *N-*glycans, and thereby inhibits virus rolling, gilding, and entry (Fig. 6D). Thus, glycan analysis of ARB plus virus infection provided crucial evidence for the antiviral activity of ARB via inhibiting the utilization of SA-linked *N-*glycans by virus. Moreover, the result provided structured information of the ARB-downregulated SA decoys, which is valuable information for developing SA receptor-targeting antiviral agents.

### ARB decreased expression levels of SA-linked glycans via inhibiting STs.

**FIG 7:**
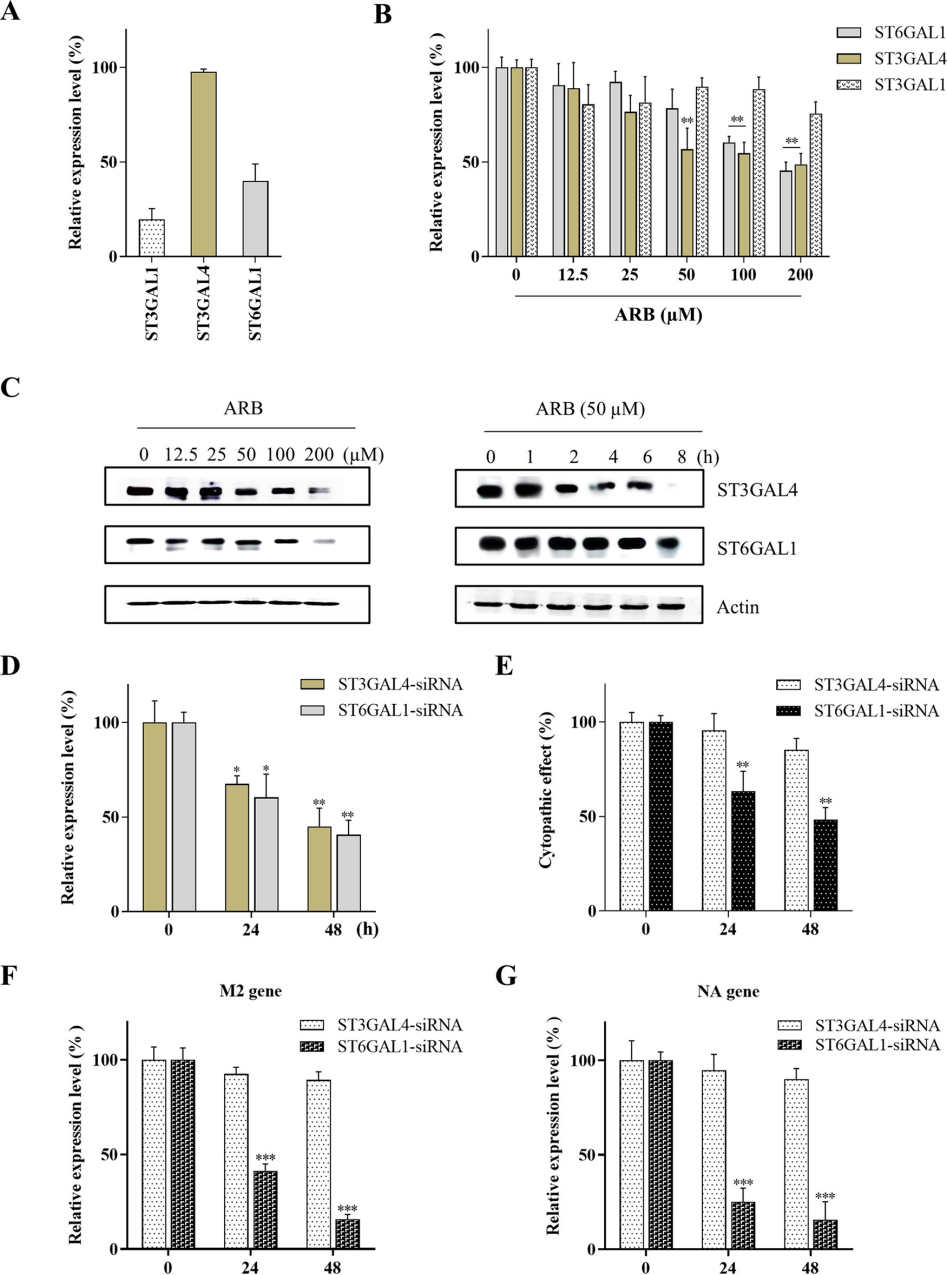
Inhibitory effect of ARB on the expression of STs. (A) The relative mRNA levels (%) of ST6GAL1, ST3GAL1, ST3GAL4 in 16-HBE cells. (B) The relative mRNA levels (%) of ST6GAL1, ST3GAL4 after ARB treatment in dose-dependent manner. (C) The protein levels of ST6GAL1, ST3GAL4 after ARB treatment were examined by using Western blot analysis. (D) The mRNA levels of ST3GAL4 and ST6GAL1 after 16-HBE cells were transfected with specific ST3GAL4 and ST6GAL1 siRNA (10 nM) for 24 h and 48 h. (E) The CPE (%) of 16-HBE cells after the cells were transfected with ST3GAL4 and ST6GAL1 siRNAs for 24 and 48 h, and then challenged with PR8 virus (MOI = 0.01). The mRNA levels of viral (F) M2 and (G) NA gene in cells after ST3GAL4- and ST6GAL1-siRNA transfected 16-HBE cells were infected with PR8 virus (MOI = 0.01). *, *P < *0.05; **, *P < *0.01; ***, *P < *0.001 compared with the control groups.

To explore the influence of ARB on STs, we monitored the expressions of three STs, ST3GAL1, ST3GAL4, and ST6GAL1, which are highly expressed in the human respiratory tract and are responsible for synthesizing *α*2,3 and *α*2,6 SA-linked glycans in mRNA level (28, 29). As shown in Fig. 7A, the expression of ST3GAL4 in 16-HBE cells was the highest and was 3- and 6-fold higher than those of ST6GAL1 and ST3GAL1, respectively. Furthermore, the mRNA level of ST3GAL4 and ST6GAL1 were consistently downregulated (*P < *0.05) following the addition of ARB for 4 h (Fig. 7B). A decrease of 50% to 60% was observed when treated with 200 μM ARB. However, the relative expression of ST3GAL1 was no different from the control group until treated with 200 μM ARB, at which point the expression was 25% lower than control group. We also examined the protein levels of ST3GAL4 and ST6GAL1 treated by ARB with varied concentrations and incubation times by using Western blot analysis (Fig. 7C), and found that ARB caused the downregulation of ST3GAL4 and ST6GAL1 at the protein level in dose- and time-dependent manner. Overall, treatment with ARB significantly inhibited expressions of two STs, ST6GAL1 and ST3GAL4, which is positively related to ARB’s capability of virus inhibition.

### Decreased level of STs and SA-linked *N-*glycans resulted in antiviral activity.

We then proposed that other approaches capable of depleting SAs would exhibit antiviral activities, just as ARB. Thus, we explored the antiviral effect of decreased levels of STs by using siRNA interference. The mRNA levels of ST6GAL1 and ST3GAL4 were decreased by 30% to 35% when ST3GAL4- and ST6GAL1-siRNAs were transfected into 16-HBE cells, and reached 50% to 60% after 48 h (Fig. 7D). As a result, the knockdown of ST3GAL4 had no significant impact on PR8 viral yield in 16-HBE cells compared with the control group. In contrast, CPE (%) was dramatically reduced in ST6GAL1 siRNA-transduced 16-HBE cells (Fig. 7E), indicating the ST6GAL1 siRNA-treated cells were protected from the PR8 virus infection. Additionally, Fig. 7F and G showed that the expressions of M2 and NA gene of PR8 virus in ST6GAL1 siRNA-treated cells reduced in a time-dependent manner, while no obvious change of M2 and NA expressions was observed in ST3GAL4 siRNA-treated cells, indicating only ST6GAL1 downregulation resulted in PR8 virus inhibition. ST6GAL1 is responsible for synthesizing *α*2,6 SA-linked glycans in hosts which are receptors preferentially recognized by human influenza virus like PR8. Thus, our results demonstrated the important role of ST6GAL1 for a successful PR8 virus infection.

Sialidase treatment was used for the removal of SAs on cell surface, sialidase S was used for the specific release of *α*2,3-linked SAs, and sialidase A can cleave both *α*2,3- and *α*2,6-linked SAs. We evaluated cell viability following treatment of two sialidases using MTT assay (Fig. S1A, B). The results indicated that treatment with sialidases showed no obvious cytotoxicity. We then monitored the altered levels of *N-*glycans in 16-HBE cells to assess the efficacy of SA removal. As shown in Fig. S1C, cells treated with sialidase S demonstrated partial desialylation; *α*2,6 SA-linked *N-*glycans remained almost unchanged, while *α*2,3 SA-linked *N-*glycans were significantly desialylated in a dose-dependent manner and generated the corresponding neutral parental *N-*glycans (for example, 5_4_0_0, 6_3_0_0) as main reaction products. Furthermore, sialidase A treatment resulted in potent desialylation of 16-HBE cells and a series of neutral *N-*glycans were detected as main reaction products. These results demonstrated the efficacy of sialidase treatment to decrease the SA-linked *N-*glycans on cell surface.

To further investigate the effect of the decreased level of SA-linked *N-*glycans on the antiviral activity, CPE inhibition assay was performed to assess PR8 viral yield in 16-HBE cells after sialidase treatment, and the cell viability of sialidase-treated cells against PR8 infection were also examined by using CCK-8 assay (Fig. S1D, E). As expected, CPE (%) of sialidase A-treated cells significantly reduced in a dose-dependent manner. On the contrary, no visible change in CPE (%) was observed in sialidase S-treated cells. Consistent with the CPE assay, CCK-8 assay showed that the cell viability was significantly increased after the treatment of sialidase A. No remarkable change of cell viability was observed in sialidase S-treated 16-HBE. All these data suggested that the decreased level of *α*2,6 SA-linked *N-*glycans resulted in PR8 virus inhibition.

### Natural ST inhibitors also exerted antiviral activity.

Three flavonoid derivatives, 3-hydroxyflavone, (+)-catechin, and (−)-epicatechin reported as natural ST inhibitors, were employed for further investigating the antiviral effect of decreased STs. Based on literature evidence, these catechin derivatives are regarded as natural ST inhibitors because of their inhibition on the ST activity, ST expression, and ST inhibition by changing conformation in binding sites of substrate (30).

The catechin derivatives were added into the 16-HBE cells for 4 h, and the drug was removed and the virus was inoculated on the treated cells. As shown in Fig. S2A, B, both mRNA levels of ST3GAL4 and ST6GAL1 were remarkably downregulated in a dose-dependent manner upon the treatment of the three compounds. We also examined the protein levels of ST3GAL4 and ST6GAL1 after the treatment of these three compounds, and found that the protein levels of STs were consistently downregulated in a dose-dependent manner (Fig. S2C). Furthermore, the cytotoxicity and antiviral activity of these three compounds are displayed in Fig. S2D, E, respectively. The results showed that all these compounds exerted potent antiviral activity against PR8 virus

### Antiviral effect of ARB was diminished upon the upregulation of STs.

**FIG 8:**
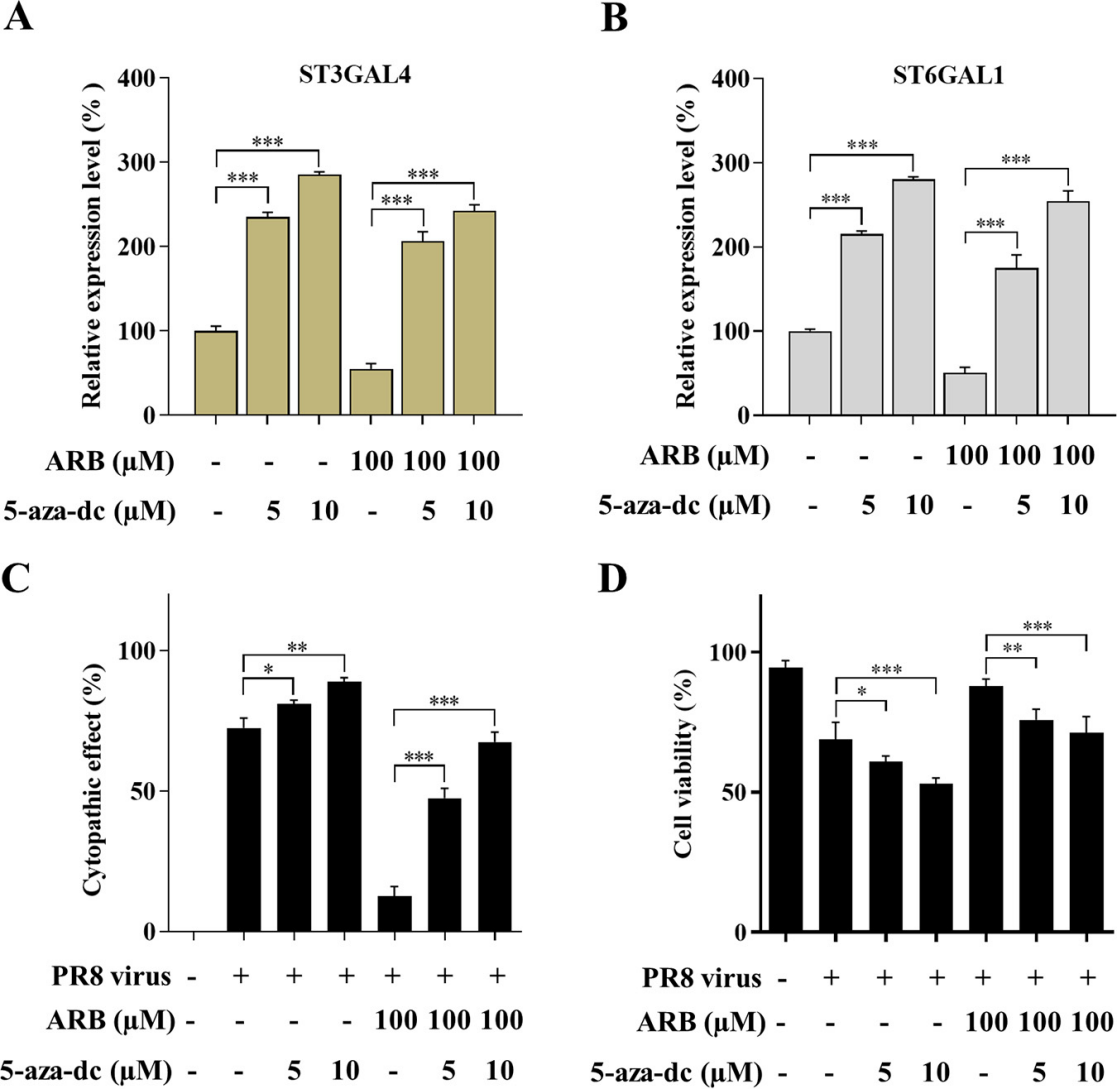
Antiviral effect of ARB with the existence of ST-inducing agent. The mRNA levels of (A) ST3GAL4 and (B) ST6GAL1 after 16-HBE cells were treated with 5-aza-dc (5,10 μM) with or without the pretreatment of ARB (100 μM). (C) The CPE (%) of 16-HBE under different treatments was as indicated. (D) The cell viability (%) of 16-HBE under different treatments by monitored using CCK-8 assay.

In order to explore the involvement of ST inhibition in the antiviral activity of ARB, 16-HBE cells were treated with a ST-inducing agent, 5-aza-dc under different conditions as indicated (31). Results showed that treatment of 5-aza-dc increased the expression levels of both ST3GAL4 and ST6GAL1 (Fig. 8A and B). We examined the effect of 5-aza-dc on the expression of STs in the presence of ARB. The results showed that 5-aza-dc increased expression of the STs in the presence of ARB in a dose-dependent manner. With the treatment of 5-aza-dc, the CPE (%) of ARB-pretreated 16-HBE cells remarkably increased and cell viability significantly reduced, indicating that the ST-inducing agent significantly diminished the antiviral effect of ARB (Fig. 8C and D). These data clearly suggested the involvement of ST inhibition in the antiviral activity of ARB.

## DISCUSSION

As members of the orthomyxoviridae family, influenza viruses are responsible for annual seasonal influenza epidemic and occasional global pandemics. Abundant glycans cover the cell surface and can be recognized by virus particles to initiate infection. This leads to interest in targeting glycosylation as a potential therapeutic approach (32). However, there is limited information of glycan structures due to challenging detection and the complexity of glycan structures. In recent years, glycomics research suggested a new diagram for the relationship among glycosylation, virulence, and receptor specificity of viruses (32). About 30 SA-linked *N-*glycans had been identified in HBE cells as complex, multi-antennas with no more than two fucoses by using a routine MALDI-TOF MS method, while rigorous analysis had not been applied to neutral *N-*glycans (33). In the present study, our application of the TiO_2_-PGC chip-Q-TOF-MS system showed great capacity of sensitivity and monitoring in detecting *N-*glycans in low level. As a result, a total of 108 *N-*glycans were authentically identified (Hex_4-10_HexNAc_2-6_dHex_0-3_NeuAc_0-3_), including 70 SA-linked *N-*glycans (from 24 compositions) and 38 neutral *N-*glycans (from 16 compositions), more than 70 *N-*glycans had not been reported previously. We have provided evidence that the existence of SA-linked *N-*glycans contained one to three fucoses and SAs, as well as glycans possessing mono-, di-, tri-, tetra-antennary, which adds credence to previous literature. The results of our study, also consistent with those by Chandrasekaran, showed that HBE cells expressed a wide range of *α*2,6 SA-linked glycans but limited *α*2,3 SA-linked glycans (33). Our results revealed the structural diversity of *N-*glycans, thus providing valuable chemical information for investigating the therapy of influenza virus infection from the point view of glycomic.

As a broad spectrum antiviral drug, ARB has been reported to exert antiviral activity against a wide range of RNA or DNA viruses from different families. Over the past decades, the underlying antiviral mechanisms of ARB have been investigated. It has been reported that ARB exhibits its antiviral activity at different steps of the virus replication cycle (34, 35). It can inhibit virus entry by interacting with virus HA and occupying a discrete binding capsule, forming an ARB-HA complex and enhancing the structural stability of HA, resulting in the failure of membrane fusion (36). ARB is a hydrophobic molecule with indole structure, which is prone to form supramolecular arrangements through the interaction with the aromatic amino acid residues of viral glycoproteins, leading to interference of membrane fusion. Additionally, 2′,5′-oligoadenylate synthase is known as an antiviral enzyme which is inhibited during virus replication, and ARB was reported to activate 2′,5′-oligoadenylate synthase, resulting in the inhibited release of viral genetic material (37). Findings also indicated that ARB can induce host cells to produce interferon, stimulate humoral responses, activate macrophage, and cause cellular and humoral immunity (36, 37). Because ARB inhibits influenza A and B virus invasion at the early stage by at least 1-h pretreatment in a one-cycle infection experiment (37), and the process of virus entry is closely associated with the glycans on hosts, we hypothesized that ARB might be capable of preventing influenza via targeting the glycans on cell surface.

Our study suggested that after being infected by the PR8 virus, the level of SA-linked *N-*glycans decreased, accompanied by an increase of neutral *N-*glycans. This is consistent with previous observations from Tatsuya Sakai and colleagues, which indicated that the NA protein on the host cell surface would cleave SAs till the suitable entry receptor showed up and bound for the virus entry (26, 27). These SA-linked *N-*glycan biomarkers might be the decoys and entry receptors of PR8. Previous studies have shown that human influenza viruses could recognize short, branched *N-*glycans as receptors, e.g., H1N1 has been reported to bind to *α*2,6 SA-linked *N-*glycans, *α*2,6/*α*2,3 di-antennary *N-*glycans, and a small amount of *α*2,3 SA-linked *N-*glycans (12, 33). Our study provided more chemical evidence for the virus binding tropism and movement pattern.

Subsequently, an extensive and remarkable downregulation of SA-linked *N-*glycans, along with an upregulation of neutral *N-*glycans, were observed upon ARB treatment. Most of the influenced SA-linked *N-*glycans were di-antennary containing one or two *α*2,6/*α*2,3-linked SAs, including some decoys and entry receptors utilized by virus. Thus, the virus utilization capability on the SA-linked *N-*glycans for rolling and entry might be impaired, which was verified by further glycan analysis with ARB plus virus infection.

Based on the biosynthesis pathway of *N-*glycans, it can be speculated that the SA-downregulating effect of ARB may ascribe to its inhibitory effect on STs, the key enzyme in the biosynthesis of SA receptors. The family of STs was responsible for initiating the sialylation process in the Golgi (38). The STs family can be classified into four groups based on the linkage type: Gal *α*2,3-STs, Gal *α*2,6-STs, GalNAc *α*2,6-STs, and *α*2,8-STs, among which Gal *α*2,3-STs catalyze the synthesis of *α*2,3 SA-linked glycans, and Gal *α*2,6-STs catalyze the synthesis of *α*2,6 SA-linked glycans (39, 40). The highest expression level of STs in human respiratory tissues are ST6GAL1 and ST3GAL4, which are closely related to the infection of influenza viruses (41–43).

To date, there has been a growing interest in targeting STs as therapeutic regimen (44). For example, treatment of SARS-infected monkeys with specific siRNAs showed the desired antiviral effect (45). Meanwhile, hexapeptide was found to act as *N*- and *O-*glycan-specific ST inhibitor for virus inhibition and cancer therapy (46). Up to now, several groups have made efforts in developing antiviral approaches based on natural and synthetic compound ST inhibitors. Our study represents the first report on the ST inhibiting effect of antiviral drugs on the market. The findings provide important information for the clinical usage of ARB, e.g., in combination with NA inhibitors to exert optimized antiviral effect. Because bacteria and some bacterial toxins could also utilize SA-linked glycans as receptors (47), the potential therapeutic value of ARB might be beyond the scope of the prevention of influenza virus infection.

In summary, we have comprehensively profiled the *N-*glycans on 16-HBE cells and revealed, for the first time, that ST inhibition and the resulted destruction of SA receptors of host cells may be an underlying mechanism for the antiviral activity of ARB. As ST inhibition has been suggested as a promising and novel antiviral approach, ARB may represent a new type of chemical entity targeting STs, and thus can be employed as template molecule for further modification.

## MATERIALS AND METHODS

### Chemicals and reagents.

ARB was kindly gifted from Guangzhou Institute of Respiratory Health (Guangzhou, China). 3-Hydroxyflavone, (+)-catechin, (−)-epicatechin, and 5-Aza-2′-deoxycytidine (5-aza-dc) were purchased from Sigma-Aldrich (St. Louis, MO, USA). ^13^C-labeled internal standards (ISs), including ^13^C-labeled 5_4_1_0, ^13^C-labeled 5_4_1_1 (sia *α*2,3), and ^13^C-labeled 5_4_1_1 (sia *α*2,6) were obtained from Asparia Glycomics (San Sebastian, Spain). Peptide *N-*glycosidase (PNGase F) was a product of New England Biolabs (Beverly, MA, USA). Glyko sialidase S and sialidase A were purchased from Prozyme (Hayward, CA, USA). Lipofectamine 3000 and TRIzol reagent were obtained from Invitrogen (Carlsbad, CA, USA), while cDNA Synthesis Kit (RR036A) and SYBR green PCR Master Mix (RR820A) were products from TaKaRa (Shiga, Japan). The CCK-8 was purchased from Dojindo Laboratories (Dojindo, Japan). Protein assay dye reagent concentrate and radioimmunoprecipitation assay (RIPA) buffer were purchased from Bio-Rad (Hercules, CA, USA) and Thermo (Waltham, MA, USA), respectively. All the primers and siRNAs used in the experiment were from BGI (Shenzhen, China). Antibodies against *β*-actin, ST3GAL4, and ST6GAL1 were from Santa Cruz Biotechnology (Santa Cruz, CA, USA). Amicon Ultra-0.5 3K centrifuge filter devices and Sep-Pak C_18_ cartridges (1 cc, 50 mg, and 55 to 105 μm) were purchased from Millipore (County Cork, Ireland) and Waters (Milford, MA, USA), respectively. Acetonitrile (ACN) of MS grade was a product from J.T. Baker (Avantor Performance Materials, LLC. Center Valley, PA, USA), while chloroform (CHCl_3_) and isopropanol (IPA) of HPLC grade were obtained from RCI Labscan Limited (Bangkok, Thailand). Ultrapure water (18.2 MΩ) was prepared using a Milli-Q system (Millipore, MA, USA).

### Cell line and virus.

Human bronchial epithelial cell line, 16-HBE, was purchased from Shanghai Institute of Biochemistry and Cell Biology (Shanghai, China) and maintained in Dulbecco’s modified Eagle’s medium (DMEM) supplemented with 10% fetal bovine serum (FBS) (Gibco), 100 U/mL penicillin-Streptomycin (Gibco), at 37°C in a 5% humidified CO_2_ incubator. Human influenza A virus/Puerto Rico/8/1934 (H1N1) (PR8) was purchased from Wuhan Institute of Virology, China Academic of Sciences. PR8 virus was incubated in 10-day-old embryonated eggs at 37°C for 48 h biosafety level 2-enhanced containment. For determination of the virus titers, the 16-HBE cell monolayer was incubated with 10-fold serial diluted serum-free virus stock solution for 2 h at 37°C. The virus inoculum was removed and replaced by serum-free DMEM, and the cells were incubated for another 48 h. An acute infection of virus was manifested by vacuolization of cells and formation of syncytia and consequently by cell lysis, which was characterized by a typical CPE. The CPE was recorded under a light microscope, and the 50% tissue culture infectious dose (TCID_50_) was calculated using the Reed-Muench method (48).

### Cytotoxic and antiviral activity assay.

The cytotoxic effects of ARB, 3-hydroxyflavone, (+)-catechin, and (−)-epicatechin on 16-HBE cells were examined applying MTT assay. Confluent monolayer of cells was incubated with indicated concentrations of compounds (ARB: 600, 900, 1,200, 1,500, and 2,100 μM; 3-hydroxyflavone, (+)-catechin, and (−)-epicatechin: 50, 100, 200, 400, and 800 μM) for 48 h. The cells were incubated with MTT solution at 0.5 mg/mL for 4 h. The supernatants were discarded, and 100 μL DMSO was added into each well to dissolve the formazan crystals. The absorbance was measured at 570 nm with a microplate reader (BioTek, Winooski, USA), and each experiment was carried out in triplicate. TC_50_ values were calculated using the Reed-Muench method (48).

The antiviral effects of ARB, 3-hydroxyflavone, (+)-catechin, and (−)-epicatechin against PR8 virus were evaluated by using CPE inhibition assay under the nontoxic concentration. Confluent monolayer of cells was incubated with indicated concentrations of each compound (12.5, 25, 50, 100, 200 μM) at 37°C for 4 h. Then inoculation supernatants were removed, and the cells were subsequently inoculated with PR8 (MOI = 0.01). After 2 h of incubation at 4°C, the supernatants were replaced with serum-free culture medium, additional incubation for 48 h at 37°C was followed. The percentage of CPE under different concentration was recorded, and each experiment was carried out in triplicate. IC_50_ (50% inhibition concentration) values of each compound were calculated using the Reed-Muench method (48).

### Capture of *N*-glycans.

In order to determine whether the *N*-glycome of 16-HBE cells are involved in the antiviral effect of ARB against PR8. The following groups were established: (i) virus group, 16-HBE cells were treated with PR8 (MOI = 0.01) at 4°C for 2 h (*n *= 8); (ii) ARB group at different concentrations, 16-HBE cells were treated with different concentrations of ARB (12.5, 25, 50, 100, and 200 μM) at 37°C for 4 h (*n *= 8 for each concentration); (iii) ARB group at different incubation times, 16-HBE cells were treated with 50 μM ARB at 37°C for different incubation times (1, 2, 4, and 8 h) (*n *= 8 for each incubation time); ARB + virus group, 16-HBE cells were treated with different concentration of ARB (12.5, 25, 50, 100, and 200 μM) at 37°C for 4 h, followed by inoculation of PR8 (MOI = 0.01) at 4°C for 2 h (*n *= 8 for each concentration). Then, *N*-glycans samples from each group of cells were prepared as described in our previous study with minor modification (24). Briefly, cells in each well were collected into 1.5 mL tubes. After rinsing three times with ice-cold PBS, the cells were lysed with 100 μl of RIPA buffer and incubated on ice for 1 h with vortex every 10 min. The samples were then centrifuged at 14,000 × *g* for 15 min at 4°C and the supernatants were subjected to buffer exchange with water and concentrated to ∼30 μL by using a 3K centrifuge filter unit. The protein concentration in each sample was determined by the Bradford assay. Next, the *N-*glycans were released by digestion with PNGase F and purified with Sep-Pak C_18_ cartridges. Samples were dried and reconstituted in 100 μL distilled water. The quality control (QC) sample was prepared by pooling all samples from different groups.

*N*-glycan profiling and quantitation was performed using our well-established TiO_2_-PGC chip MS method (24). In brief, the chromatographic separation of *N*-glycans was carried out on an Agilent 1260 Infinity HPLC Chip LC system (Agilent, Santa Clara, CA, USA) with a customized TiO_2_-PGC chip (Agilent, Waldbronn, Germany). The MS and MS/MS analysis of *N*-glycans were performed on an Agilent 6550 iFunnel quadrupole time-of-flight (Q-TOF) MS in positive mode, whereas the quantitation of *N*-glycans was acquired on an Agilent 6490 iFunnel triple quadrupole (QQQ) MS in MRM mode.

The SA linkage of *N*-glycans were further identified by using sialidase reaction. Briefly, 2 μL of sialidase A or sialidase S was incubated with 14 μL of pooled sample and 4 μL 5× reaction buffer in a 20 μL reaction. The reaction mix was incubated at 37°C for 1 h followed by MS analysis.

### Western blot analysis.

16-HBE cells were treated with ARB, 3-hydroxyflavone, (+)-catechin, and (−)-epicatechin at indicated concentrations (12.5, 25, 50, 100, 200 μM) or treated with ARB at different incubation times (1, 2, 4, 6, 8 h) at 37°C for 4 h, the relative protein levels of STs were detected by using Western blot analysis. In brief, cells were washed with cold PBS for three times and lysed in RIPA buffer (containing protease/phosphatase inhibitors). After the protein concentrations were determined by using BCA protein assay (Pierce, Rockford, IL, USA), the absorbance of each sample was measured at 595 nm with a microplate reader (BioTek, Winooski, USA), then equal amounts of proteins (80 μg) were subjected to sodium dodecyl sulfate-polyacrylamide gel electrophoresis (SDS-PAGE) and transferred on a nitrocellulose membrane. The nitrocellulose membrane was incubated with specific primary antibodies at 4°C overnight, followed by washing with PBS-T for three times. Then it was incubated with IRDye 800-labeled secondary antibodies (LI-COR Biosciences, Lincoln, NE, USA) at room temperature for 1 h. The detection was performed by the Odyssey Infrared Imaging System (LI-COR Biosciences).

### RNA isolation and RT-PCR analysis.

16-HBE cells were incubated with indicated concentrations of ARB, 3-hydroxyflavone, (+)-catechin, and (−)-epicatechin (12.5, 25, 50, 100, 200 μM) at 37°C for 4 h and the relative levels of ST3GAL4, ST6GAL1 were monitored by using RT-PCR. Total RNA was extracted using TRIzol reagent, followed by the addition of 200 μL of CHCl_3_. The mixture was centrifuged at 14,000 × *g* for 15 min at 4°C and the aqueous phases was carefully collected into a new tube. Next, RNA was precipitated from the aqueous phase with equal volume of IPA. After centrifugation at 14,000 × *g* for 15 min at 4°C, the RNA pellet was washed with 75% ethanol, air-dried, and dissolved in an appropriate amount of diethyl pyrocarbonate-treated (DEPC) water. The concentration and quality of total RNA was measured by using a NanoDrop spectrophotometer (Thermo, Wilmington, DE, USA).

Total RNA was reverse transcribed using the cDNA Synthesis Kit according to the manufacturer’s instructions. RT-PCR was performed using SYBR green PCR Master Mix with an ABI ViiA 7 real-time PCR system (Applied Biosystems, Foster City, CA, USA). The relative expression levels of ST3GAL1, ST3GAL4, ST6GAL1, viral M2, and NA gene were calculated using the 2^−ΔΔCt^ method with GAPDH as an internal reference gene. The following primers were used: ST3GAL1, 5′-GCTCTTGGAGGACGACACCTACCGATG-3′ (forward) and 5′-CCACATTCCCAGGCACCACTCTGAACA-3′ (reverse); ST3GAL4, 5′-AACAACCCAGACACACTCCTCGTCCTG-3′ (forward) and 5′-ACCCTTTCGCACCCGCTTCTTATCACT-3′ (reverse); ST6GAL1, 5′-GGCAGGTGTGCTGTTGTGTCGTCAG-3′ (forward) and 5′-ATCTTGTTGGAAGTTGGCTGTGGGTGC-3′ (reverse); NA, 5′-ACATCTGCAGTGGGGTTTTC-3′ (forward) and 5′-ACCAATCAGTCATTGCCACA-3′ (reverse); M2, 5′-GGGAAGAACACCGATCTTGA-3′ (forward) and 5′-GCAAGTGCACCAGCAGAATA-3′ (reverse); GAPDH, 5′-GCTCTCCAGAACATCATCCCTGCCTCT-3′ (forward) and 5′-CGACGCCTGCTTCACCACCTTCTTG-3′ (reverse).

### ST3GAL4 and ST6GAL1 gene silence and activation.

Gene expressions of ST3GAL4 and ST6GAL1 in 16-HBE cells were silenced by transfecting siRNA (10 nM) using Lipofectamine 3000 according to the manufacturer’s protocol. The following siRNA sequences were used: ST3GAL4, 5′-UGAGAUCGUAUGAAUGACU-3′ (forward) and 5′-ACUCUAGCAUACUUACUGA-3′ (reverse); ST6GAL1, 5′-GUACCAGAAUCCGGAUUAU-3′ (forward) and 5′-CAUGGUCUUAGGCCUAAUA-3′ (reverse). The relative levels of ST3GAL4 and ST6GAL1 after 24 h or 48 h transfection were determined by using RT-PCR analysis. The effect of ST3GAL4 and ST6GAL1 gene silencing against PR8 infection (MOI = 0.01) in 16-HBE cells were further evaluated by using CPE inhibition assay, and the M2 and NA gene of PR8 virus in ST3GAL4- and ST6GAL1-siRNA transfected cells was evaluated by using RT-PCR analysis.

Gene expressions of ST3GAL4 and ST6GAL1 in 16-HBE cells were activated by incubating the cells with indicated concentrations of 5-aza-dc (5, 10 μM) every 24 h for 3 days, and the relative ST3GAL4 and ST6GAL1 gene levels with or without the treatment of ARB (at 100 μM for 4 h treatment) were determined by using RT-PCR analysis. The effect of ST3GAL4 and ST6GAL1 gene activation (under 5 or 10 μM 5-aza-dc) to the antiviral effects of ARB (at 100 μM for 4 h pretreatment) against PR8 were evaluated by using CPE inhibition assay and CCK-8 assay.

### Sialidase treatment of 16-HBE cells.

*α*2,3 SA or *α*2,6 SA on 16-HBE cells were removed by incubating the cells with 100 U/mL of sialidase A or sialidase S for 0.5, 1, 1.5, and 2 h, as well as incubating the cells with 50, 100, and 200 U/mL of sialidase A or sialidase S, respectively for 2 h. The cytotoxic effects of different incubation times and concentrations of sialidase treatment were determined by using MTT assay. In addition, the efficacy of sialidase treatment were evaluated by using glycomic analysis, and the effects of sialidase treatment to the *N*-glycome of 16-HBE cells and against the PR8 infection (MOI = 0.01) in 16-HBE cells were evaluated by using CPE inhibition assay and CCK-8 assay.

### CCK-8 viability assay.

The viability of sialidase-treated 16-HBE cells against PR8 and the effect of ST gene activation to the antiviral activity of ARB was monitored by using CCK-8 assay. Briefly, 16-HBE cells were treated under different conditions as indicated above. Then the supernatants were replaced with serum-free culture medium for 48 h of incubation at 37°C. Thereafter, 16-HBE were added with 10 μM CCK-8 solution and incubated for 1 h at 37°C. The absorbance at 450 nm was measured to evaluate cell viability using an automated microplate reader (Thermo Fisher Scientific, Waltham, MA, USA).

### Statistical analysis.

All MS and MS/MS data were analyzed by Agilent MassHunter Qualitative Analysis B.06.00 software as described in our previous study (24). Find by formula (FBF) algorithm and our established personal *N*-glycan database (consisting of 3,795 *N-*glycan compositions) were utilized for mining *N*-glycans in 16-HBE cells. MRM data were processed by Agilent MassHunter Quantitative Analysis B.06.00 software. The level of each *N-*glycan in 16-HBE cells was quantified based on peak area ratio of *N-*glycan against its corresponding IS, in which ^13^C-labeled 5_4_1_0, ^13^C-labeled 5_4_1_1 (sia *α*2,3), and ^13^C-labeled 5_4_1_1 (sia *α*2,6) were used as the calibration IS for neutral *N*-glycans, SA-linked *N*-glycans with sia *α*2,3 linkage, and SA-linked *N*-glycans with sia *α*2,6 linkage, respectively.

Multivariate analysis was performed by SIMCA version 15.0.2 (Sartorious Stedim Biotech, Umea, Sweden). Statistical significances were calculated using GraphPad Prism 6 (GraphPad Software, La Jolla, CA, USA), a two-sided *P value* of < 0.05 was considered statistically significant. The software GraphPad Prism 8 (GraphPad Software, La Jolla, CA, USA) was used for heatmap analysis. Continuous variables were presented as mean ± SD, and the differences between the groups with *t* test analysis or Mann-Whitney U test. *P < *0.05 was considered as statistically significant.
