# Supplementary material for: Glycomic Analysis Reveals That Sialyltransferase Inhibition Is Involved in the Antiviral Effects of Arbidol
Source: J Virol. 2022 Mar 23;96(6):e02141-21. doi: 10.1128/jvi.02141-21 (PMC8941891; doi:10.1128/jvi.02141-21)
Supplement: Supplemental file 1 — Fig. S1 and S2. Download jvi.02141-21-s0001.pdf, PDF file, 0.9 MB [file jvi.02141-21-s0001.pdf]

SUPPORTING INFORMATION

Table S1. The MS, MS/MS data and MRM transitions of the 108 *N*-glycans identified in 16-HBE cells.

Fig. S1 Evaluation of PR8 viral yield in 16-HBE cells after sialidase treatment.

Fig. S2 The antiviral activity of natural ST inhibitors.

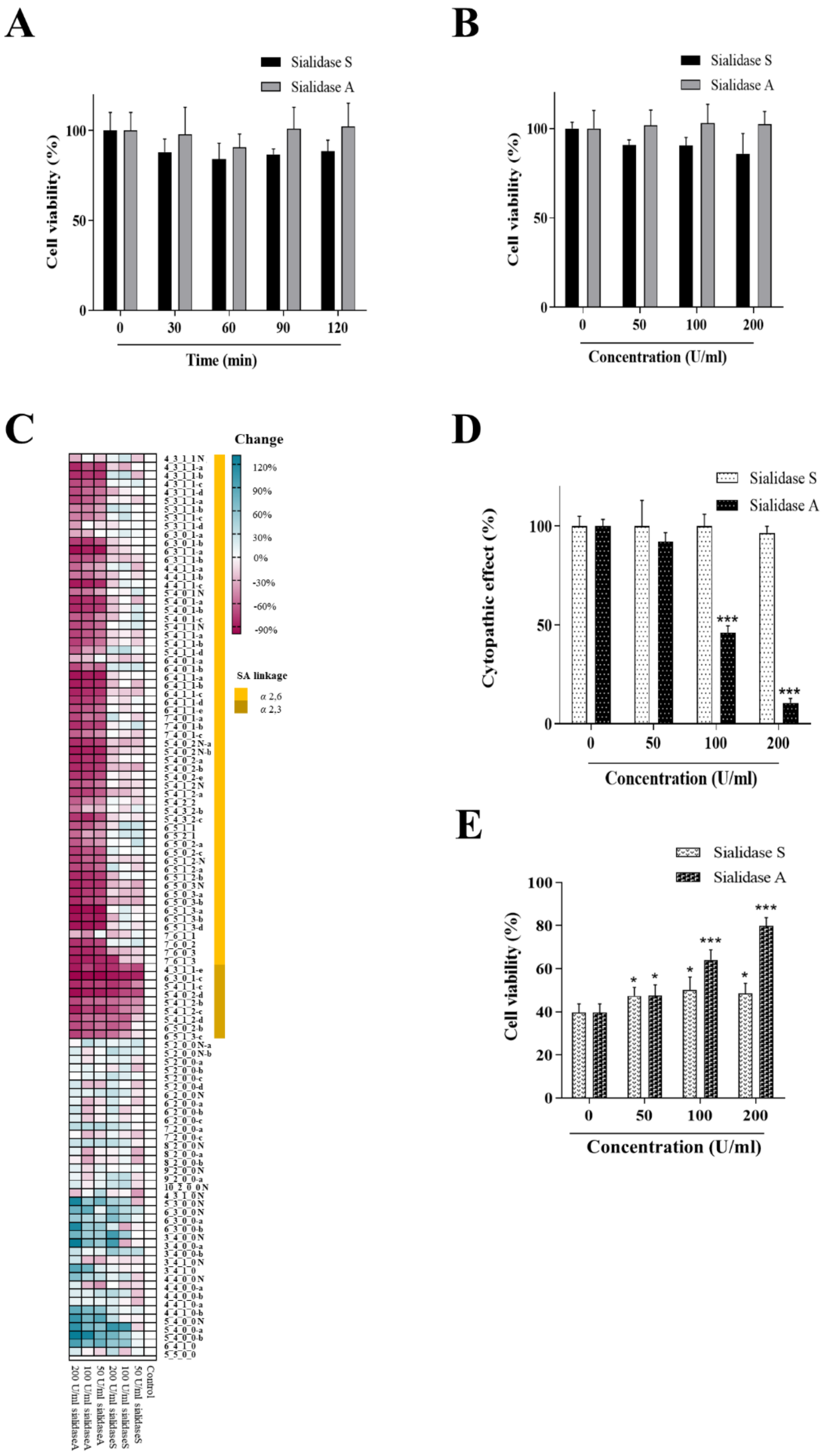

Fig. S1 Evaluation of PR8 viral yield in 16-HBE cells after sialidase treatment. 16-HBE cells were treated with sialidase A or S in a (A) time- or (B) dose-dependent way

as indicated, cell viability (%) was monitored by using MTT assay. (C) Heatmap displays the altered levels of SA-linked *N*-glycans and neutral *N*-glycans in 16-HBE cells after 2 h treatment of sialidase A and S (0, 50, 100, 200 U/ml). (D) 16-HBE cells were treated with sialidase A or S for 2 h, then cells were challenged with PR8 virus (MOI = 0.01), the CPE (%) was quantified. (E) 16-HBE cells were treated with sialidase A or S at different concentrations for 2 h, then cells were challenged with PR8 virus (MOI = 0.01), the cell viability (%) was monitored by using CCK-8 assay. *$p < 0.05$; **$p < 0.01$; ***$p < 0.001$ compared with the control groups, the data form were quantified and represented as the mean ± SD of three independent experiments.

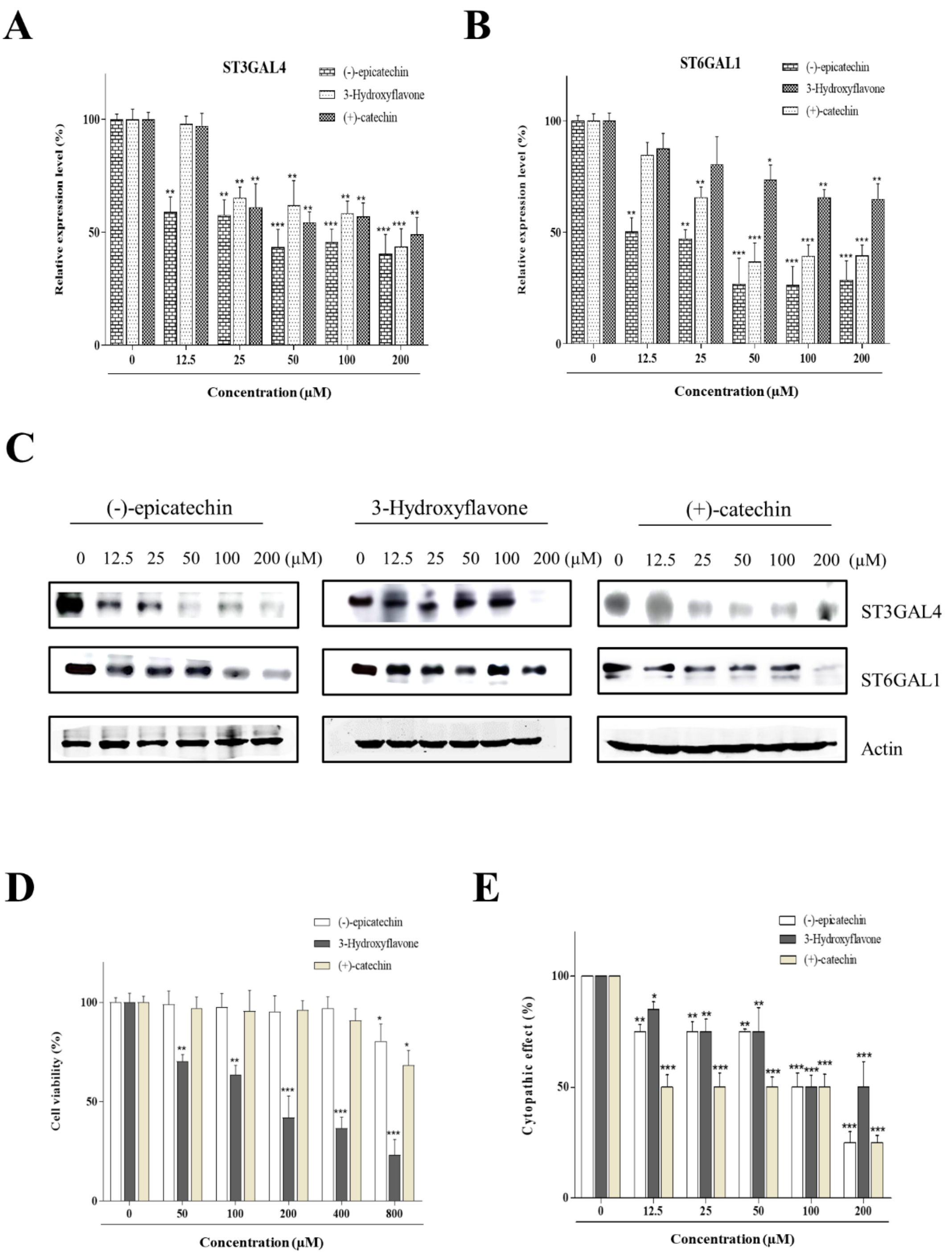

Fig. S2 The antiviral activity of natural ST inhibitors. 16-HBE cells were treated with three natural ST inhibitors, 3-hydroxyflavone, (+)-catechin and (-)-epicatechin in a dose-dependent way as performed, mRNA levels of (A) ST3GAL4 and (B) ST6GAL1 were quantified by using RT-PCR. (C) The protein levels of ST6GAL1, ST3GAL4 after

the treatment of three natural ST inhibitors were examined by using western blot. (D) Cell viability (%) after the treatment of three natural ST inhibitors was monitored by using MTT assay. (E) Inhibitory effect of three compounds against PR8 virus (MOI = 0.01) in 16-HBE cells was monitored using CPE assay. $^{*}p < 0.05$; $^{**}p < 0.01$; $^{***}p < 0.001$ compared with the control groups.
